# Amyloid-β 1-42 fibrils regulate SH-SY5Y cell adhesion in a delayed manner

**DOI:** 10.1177/13872877261429855

**Published:** 2026-03-18

**Authors:** Urša Pečar Fonović, Slavko Kralj, Janko Kos

**Affiliations:** 1 63721 Faculty of Pharmacy, University of Ljubljana, Aškerčeva, Slovenia; 2 Department for Materials Synthesis, 61790 Jožef Stefan Institute, Ljubljana, Slovenia; 3 Nanos SCI, Nanos Scientificae d.o.o, Ljubljana, Slovenia; 4 Department of Biotechnology, 61790 Jožef Stefan Institute, Ljubljana, Slovenia

**Keywords:** Alzheimer's disease, amyloid-β fibrils, cell adhesion, SH-SY5Y

## Abstract

Amyloid-β peptide (Aβ), a hallmark peptide in the pathology of Alzheimer's disease, together with the amyloid-β protein precursor, is increasingly associated with the disruption of cell adhesion. In addition to its well-characterized role in plaque formation and synaptic dysfunction, Aβ interacts with various adhesion molecules and extracellular matrix components, thereby impairing neuronal connectivity and integrity. We have shown that pretreatment of SH-SY5Y cells with Aβ_42_ fibrils affects cell adhesion; however, we did not observe this effect with Aβ_42_ monomers. Understanding the molecular mechanisms by which Aβ fibrils disrupt cell adhesion pathways may reveal new therapeutic approaches to prevent disease progression.

## Introduction

Alzheimer's disease (AD) is a neurodegenerative disorder characterized by two distinctive features, the extracellular plaque deposits of amyloid-β (Aβ) and the hyperphosphorylation of tau protein aggregated intracellularly as neurofibrillary tangles.^1^ Aβ is derived from amyloid-β protein precursor (AβPP), a transmembrane protein involved in various processes such as neuronal development, neurite outgrowth, signal transduction, synaptogenesis, axonal protein trafficking, cell adhesion, etc.^2,3^ Several Aβ species are generated from AβPP by proteolytic processing by β- and γ-secretases.^2^ γ-secretase usually produces Aβ_40_, but, in neuritic plaques Aβ_42_ is most abundant, and occurs mainly in a filamentous form.^1,4^ Physiological functions have been demonstrated for low levels of Aβ. Plant et al. treated various neuronal cells (rat cortical neurons and cerebellar granule neurons as well as the SH-SY5Y cell line) with β- and γ-secretase inhibitors, which elicited cell death. The same result was obtained when endogenous Aβ was neutralized with a specific antibody. Cell viability was rescued by the addition of exogenous Aβ_40_ and, to a lesser extent, Aβ_42_ at picomolar concentrations. However, the viability of other cell types, such as astrocytes, was not affected by secretase inhibitors.^5^ It has also been shown that Aβ has a modulatory role in neurotransmission and memory.^6^ There is only a weak correlation between the level and distribution of Aβ and the clinical expression of AD. Therefore, Aβ may trigger the events in the early stages of AD that may proceed independently of Aβ and even in its absence.^1,7^ Another possible role of Aβ could be associated with cell adhesion processes as it contains a RHD (arginine-histidine-aspartic acid) triplet that resembles the RGD (arginine-glycine-aspartic acid) motif, a motif recognized by integrins.^8,9^ Several groups have shown that different integrins interact with Aβ monomers, oligomers and fibrils.^10^ In this study we investigated whether Aβ_42_ monomers and Aβ_42_ fibrils influence SH-SY5Y cell adhesion also after Aβ removal from cell culture.

## Methods

### Cell culture

Human neuroblastoma cell line SH-SY5Y was obtained from the ATCC (Cat. No. CRL-2266, American Type Culture Collection). The cells were grown in complete medium: Advanced DMEM (Cat. No. 12-491-015, Fisher Scientific, MA, USA) supplemented with 10% FBS, 1% penicillin/streptomycin and 1% L-glutamine. For the treatment of the cells with Aβ_42_ (cytotoxicity assay and cell pretreatment for the adhesion assay) a reduced culture medium containing only 2% FBS was used.

### Peptide preparation

Lyophilized Aβ_42_ peptide (Cat. No. A-1163-2, rPeptide, GA USA) was dissolved in dimethyl sulfoxide (DMSO) to a concentration of 5 mM. Different forms of the peptide (monomers or fibrils) were prepared as previously described.^11^ Briefly, to prepare monomers, the peptide was diluted to the desired concentration with reduced culture medium just before use. To prepare fibrils, the peptide was first diluted in 10 mM HCl to a concentration of 100 µM and incubated for 24 h at 37°C, and then further diluted to the desired concentration with reduced culture medium. Appropriate controls were prepared in the same way using DMSO instead of the peptide. The final DMSO concentration in cell assays was 0.04%.

### Transmission electron microscopy (TEM)

The morphology of the nanostructure of monomers and fibrils was determined at a concentration of 100 μM using a transmission electron microscope. First, 20 μL of the fibril or monomer solution was pipetted onto a copper-carbon coated TEM grid, and then the grid was air-dried. TEM images were obtained with a Jeol JEM 2100 instrument at 100 kV.

### Cytotoxicity assay

A 96-well plate was seeded at 4 × 10^4^ cells/well and cultured for 24 h. The cells were then treated with different concentrations of Aβ_42_ (10 nM to 4 µM) in the form of monomers or fibrils diluted in a reduced culture medium for 24, 48 or 72 h. Possible cytotoxic activity was determined by adding the MTS reagent (CellTiter 96 Aqueous One Solution Cell Proliferation Assay, Promega, WI, USA) and measuring A_492_ after 70 min. The percentage of viable cells was calculated relative to the control cells (cells treated with DMSO or DMSO and 10 mM HCl in a reduced culture medium for monomers and fibrils, respectively), which were considered 100% viable. Two independent biological experiments were performed, each in quadruplicate.

### Real-time cell adhesion assay

A 6-well plate was seeded at 8 × 10^4^ cells/ml. After 48 h, cells were treated with 2 µM Aβ_42_ monomers, fibrils, or control medium for 24 h. Cells were then washed with PBS, detached, and washed with PBS again.

Cell adhesion was measured using the Real-Time Cell Analyzer Dual Plate (RTCA DP) of the xCELLigence system (Agilent, CA, USA). The device monitors cell adhesion in real time and records cell responses throughout the course of the experiment. 100 µL of the complete culture medium was pipetted into the wells of the E-plate (Cat. No. 300600890, Agilent, CA, USA), and incubated on the device for 30 min at 37°C. After measuring the background, 100 µL of cells (2 × 10^4^ cells/well) were added, that had previously been exposed to a sub-toxic concentration of Aβ_42_ monomers or fibrils for 24 h and washed with PBS. In a control experiment, the same concentration of control cells was added to the wells. The measured cell index (CI) represents the relative change in electrical impedance which indicates the cell's status. The dynamic CI values were monitored at 8-min intervals from the time the cells were added until the end of the experiment (24 h). The measurements were analyzed using the RTCA software and the results were normalized to the control cells, which were assigned 100% adhesion.

## Results

### Determination of the sub-toxic concentration of Aβ_42_ for Sh-SY5Y cells

**Figure 1.**
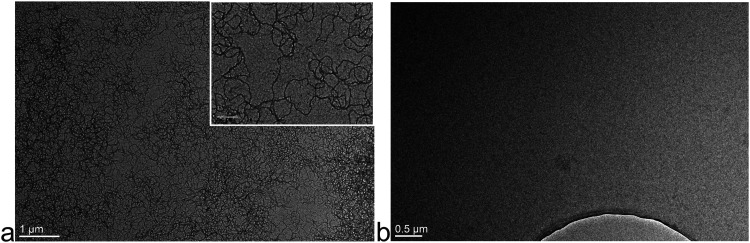
Fibrils (a) and monomers (b) of Aβ_42_. The different forms of the peptide were imaged by TEM at a concentration of 100 µM. The bar size is 1 µm in a or 0.2 µm in a (insert), 0.5 µm in b.

The sub-toxic concentration of the Aβ_42_ was determined in order to perform further experiments without cytotoxic effects on the cells. First, the efficacy of our method to prepare monomers and fibrils of Aβ_42_ was verified by TEM. The presence of fibrils was shown at 100 µM peptide concentration (Figure 1(a)), which were further diluted for use. No fibrils were present in the monomer sample of Aβ_42_ (Figure 1(b)).

**Figure 2.**
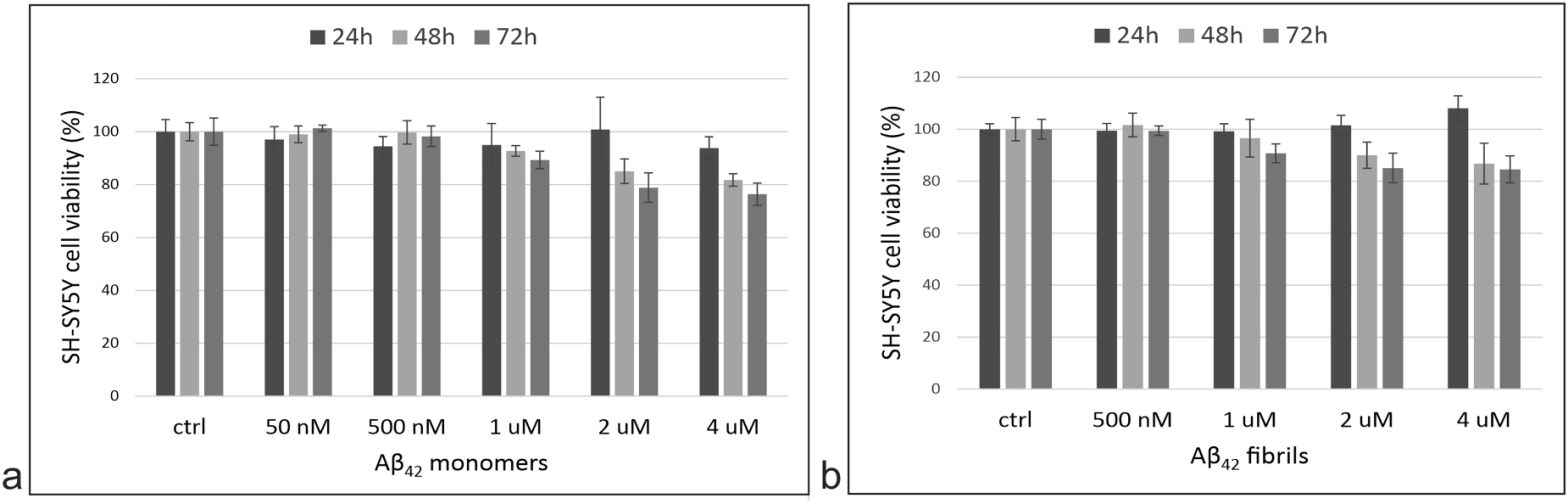
Aβ_42_ cytotoxicity. SH-SY5Y cells were exposed to different concentrations of Aβ_42_ monomers (a) and fibrils (b) for 24, 48, or 72 h. The cytotoxic effect was determined by adding MTS reagent and measuring the absorbance at 492 nm after 70 min. Each experiment was performed twice in quadruplets.

Next, the cells were exposed to different concentrations of monomers or fibrils and cytotoxicity was determined using the MTS assay. An Aβ_42_ concentration of 2 µM was determined to be sub-toxic for 24-h experiments. For 48- and 72-h experiments, the optimal concentration was 500 nM (Figure 2). In general, with longer incubation times or at higher concentrations, Aβ_42_ monomers are slightly (5–10%) more cytotoxic to SH-SY5Y cells than the fibrils.

### Fibrils of the Aβ_42_ reduce cell adhesion

**Figure 3.**
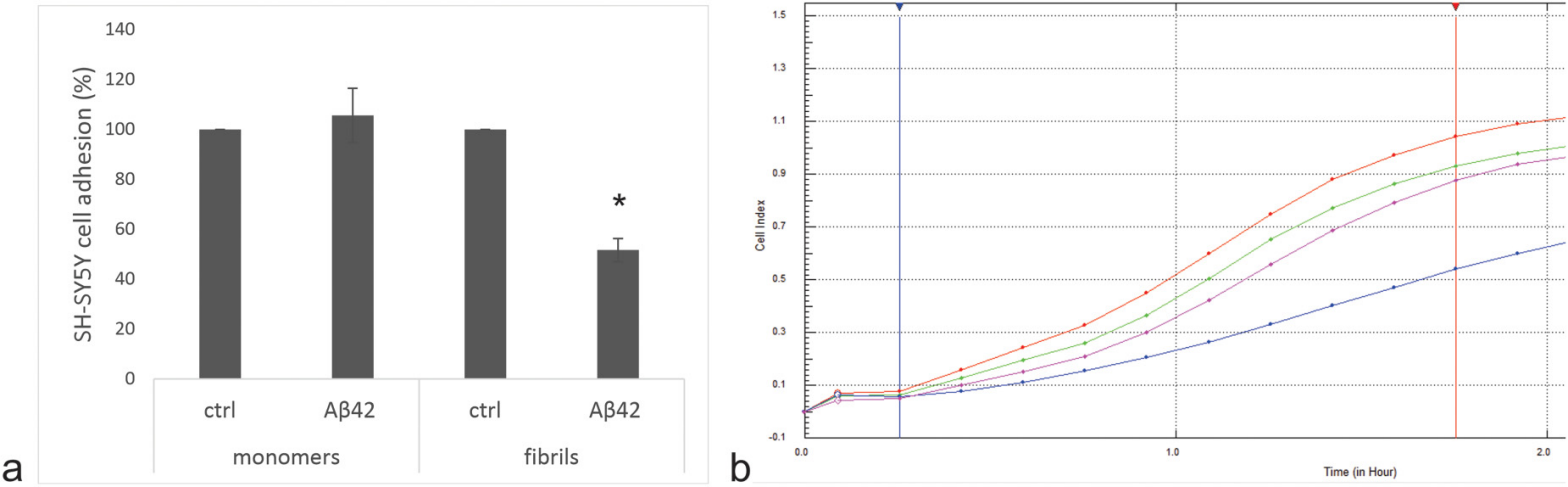
Aβ_42_ fibrils affect the adhesion of sh-SY5Y cells. The adhesion assay was performed using the xCELLigence system. a) The level of cell adhesion was assessed by the increase in curve slopes (1/h). Results are normalized to control cells with assigned 100% adhesion. b) Representative adhesion curves of cells pretreated with Aβ_42_ monomers (green), Aβ_42_ monomer control (red), Aβ_42_ fibrils (blue) and Aβ_42_ fibril control (purple) are shown. The slopes were calculated between the blue and red vertical lines. Assays were performed twice, each time in triplicate. The results are mean values ± stdev. *p = 0.03 (T-test). (Color figure available online).

SH-SY5Y cells were exposed to a sub-toxic concentration (2 µM) of Aβ_42_ monomers or fibrils for 24 h. The cells were then washed with PBS and cell adhesion to the xCELLigence E-plates was measured in real time using the RTCA-DP device. The cells adhered to the surface within the first 100 min. We showed that the cell adhesion of the cells pretreated with the fibrils was reduced by approximately 40%, while no difference was observed with the monomers (Figure 3), indicating that the monomers do not cause such a delayed effect on cell adhesion.

## Discussion

Various diseases, including AD, are associated with amyloids, highly ordered peptide or protein aggregates that are rich in cross-β sheet structures.^12^ Amyloid fibrils in general, including Aβ_42_ fibrils, have been shown to be involved in several cell functions, including cell adhesion and cell spreading. Cell adhesion to the extracellular matrix (ECM) is a prerequisite for various processes such as cell differentiation, proliferation and survival,^13^ and there are several studies describing the involvement of Aβ in cell adhesion. The studies differ in the substrate used in their experimental setup, using Aβ and components of the ECM as well as non-coated plastic plates to test cell adhesion. SH-SY5Y cells were shown to adhere to an Aβ_40_- and an Aβ_42_-coated surface.^8,9,13^ Postuma and co-workers grew primary neurons on Aβ as a substrate. A low concentration of Aβ stimulated neurite outgrowth while a higher concentration led to a reduction in cell adhesion, followed by inhibition of neurite outgrowth.^14^ The effect was demonstrated for Aβ_40_ fibrils and for Aβ_42_ fibrils and monomers, and the authors suggested that increasing Aβ concentrations may prevent normal cell-cell and cell-ECM adhesion, causing brain dysfunction.^1^ Chen and Yankner treated different cell types with an antibody specific for the first 16 amino acid residues of the Aβ using poly-L-lysine coated plates or without coating. In differentiated rat pheochromocytoma PC-12 cells, retraction of the neurites was observed, leading to cell detachment. A similar result was demonstrated for various neuronal and non-neuronal cells.^15^ It was also shown that the attachment of SH-SY5Y cells to the fibronectin-coated surface was dose-dependent, with more cells attaching at higher fibronectin concentrations. The attachment decreased after the cells were exposed to an Aβ consisting of the first 16 amino acid residues of the Aβ_42_, which also contained a RHD sequence. This result suggested that the interaction between the cell and Aβ_42_ is integrin-dependent, as confirmed by an anti-β1 integrin antibody that blocked cell attachment to both fibronectin- and Aβ_16_ -coated surfaces.^8^ Next, Matter and co-workers reported that Aβ_40_ oligomers bind to α5β1 integrin via the RHD sequence, after which they are internalized and degraded. A consequence of this increased clearance of soluble Aβ is reduced formation of insoluble Aβ.^9^ In a study by Woo and co-workers, soluble Aβ_42_ oligomers were found to deplete membrane levels of β1 integrin on primary cortical and hippocampal neurons.^16^

In this research, we used a different experimental setup to study the adhesion of SH-SY5Y. We investigated whether the Aβ_42_ can affect the adhesion of SH-SY5Y cells after the peptide is removed from the cell culture. We examined the adhesion of cells that had been pretreated with Aβ_42_ for 24 h and then allowed to adhere to a polyethylene terephthalate sensor substrate material without the presence of Aβ_42_. The peptide was used in both monomeric and fibrillar forms at a sub-toxic concentration of 2 µM. Using the xCELLigence system, which allows us to follow the process of cell adhesion in real time, we observed a 40% decrease in cell adhesion in the first 2 h after 24 h of exposure of cells to Aβ_42_ fibrils. At the same time, cells previously exposed to Aβ_42_ monomers showed no differences in cell adhesion compared to control cells. This means that Aβ_42_ fibrils can exert a delayed effect on the cell adhesion.

Prefibrillar aggregates have also been found to be able to interact with the cell membrane leading to numerous events, including the loss of certain membrane-bound protein functions.^17,18^ Zhu and co-workers treated cells with globular and non-fibrillar Aβ_40_, resulting in cytoskeletal reorganization, loss of cell contacts, etc.^17^ The tachykinin or neurokinin NK1 receptor is a possible candidate for interaction with Aβ. It is a G-coupled receptor found in the central and peripheral nervous system. Together with its primary ligand, substance P, it is involved in neuroinflammation, which plays an important role in the progression of AD.^19^ NK-1 receptor-mediated effects have been shown to influence the expression of endothelial adhesion molecules, with the expression of ICAM, VCAM and other adhesion molecules increasing following receptor stimulation in vascular endothelial cells, dermal endothelial cells and cerebral vessels.^19–22^ Amino acid residues 25–35 of the Aβ are homologous to the tachykinin family and can therefore interact with the NK1 receptor, which is also expressed by SH-SY5Y cells. The effects of such an interaction have been mimicked by tachykinin antagonists.^23,24^ Our results suggest that Aβ_42_ fibrils bind to a cell receptor such as the NK1 receptor as an antagonist, causing the decrease in adhesion molecule expression that leads to the observed delayed effect of Aβ_42_ fibrils on SH-SY5Y adhesion. Further studies will be required to elucidate this effect and identify the receptors involved.
